# Orbital Myiasis

**Published:** 2011-07

**Authors:** Gholamreza Khataminia, Roja Aghajanzadeh, Babak Vazirianzadeh, Mahmoud Rahdar

**Affiliations:** Jundishapur University of Medical Sciences, Ahvaz, Iran

**Keywords:** Myiasis, Orbit, *Chrysomya bezziana*

## Abstract

**Purpose:**

To present a case of massive orbital myiasis.

**Case Report:**

An 87-year-old debilitated woman suffering from left ocular pain of four days’ duration presented with a severely necrotized left orbit and several attached live larvae. The upper and lower eyelids and the eyeball were completely destroyed. She had history of eyelid surgery in the same eye due to a skin lesion, apparently some type of skin cancer, 15 years before. The larvae were identified as *Chrysomya bezziana* (Diptera: Calliphoridae) or old world screwworm fly.

**Conclusion:**

Infestation of ocular and orbital tissues by fly larvae (ophthalmomyiasis) progresses rapidly and can completely destroy orbital tissues within days, especially in patients with poor general health. Treatment consists of removal of the larvae and surgical debridement.

## INTRODUCTION

Infestation of ocular and orbital tissues with fly larvae (ophthalmomyiasis), is a rare condition that occurs throughout the world. Affected patients are usually old and suffer from debilitating underlying conditions. Once established, myiasis progresses rapidly and can completely destroy orbital tissues within days.

## CASE REPORT

**Figure 1:**
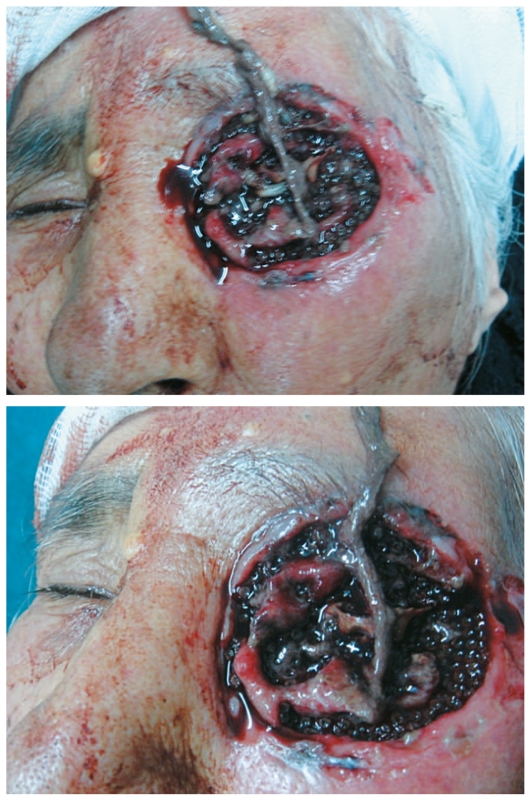
Initial presentation of the patient

An 87-year-old woman was referred to our hospital in May 2010 with severe left ocular pain since 4 days before. On initial examination, the left orbit was severely necrotized with several live larvae attached to the necrotic tissue. The upper and lower eyelids and the eyeball were completely destroyed. Periorbital tissues were swollen and erythematous (Fig. 1).

According to her grandchild, the patient had not been able to walk or sit for several years, presumably due to back injury. She also had bladder and bowel incontinence. However, her mental status was intact. She had history of eyelid surgery due to a skin lesion 15 years ago which had been confirmed to be skin cancer. There were no medical records or documents however, and she had experienced no recurrences ever since.

Orbital CT scan revealed disorganized globe and periocular tissues, with hemorrhagic patches in the left orbit filled with larvae. The orbital walls seemed intact and no larvae could be detected in the periorbital sinuses or cranium.

**Figure 2:**
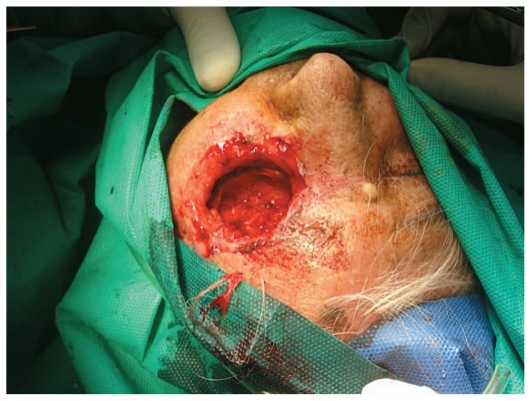
Appearance after exenteration

**Figure 3:**
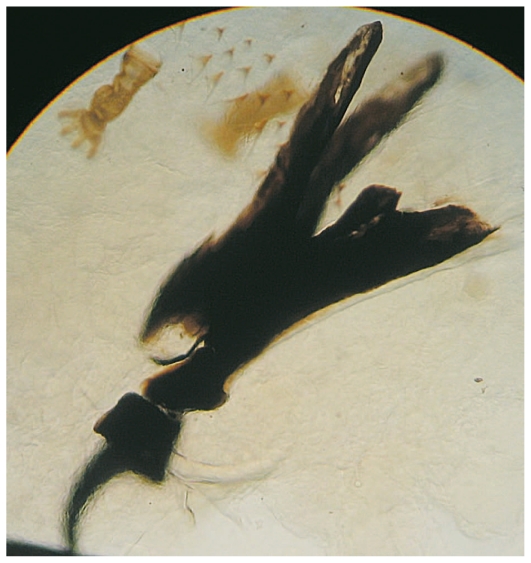
Cephalopharyngeal skeleton and anterior spiracle

**Figure 4:**
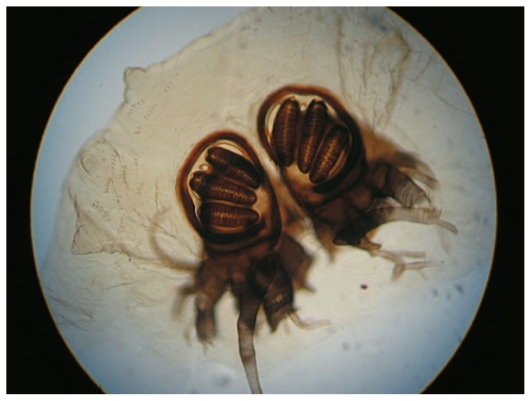
Posterior spiracles

**Figure 5:**
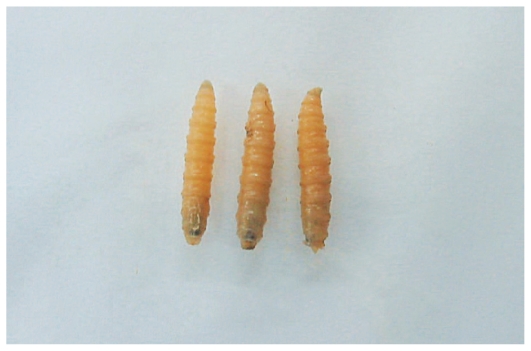
Third instar of *Chrysomya bezziana* larvae

On the day of admission, the patient underwent exenteration of the left orbit (Fig. 2). All necrotic tissues and over 150 larvae were removed. A few larvae were collected and preserved in 70% ethanol. The larvae were identified as *Chrysomya bezziana* (Diptera: Calliphoridae) or old world screwworm fly. The most distinctive characteristics of this species include one pair of anterior spiracles with 4 to 6 digit projections at the ends, located on the first segment of the third instar of the larvae above the cephalopharyngeal skeleton (one pair of hooks at the base) (Fig. 3), and one pair of posterior spiracles at the bottom of the third instar of the larvae. Each is surrounded by a complete peritreme, without a distinct button. There are 3 slits inside each peritreme, which continue to its end (Fig. 4). Figure 5 is a picture of the complete third instar larva of *Chrysomya bezziana*, removed from the patient’s orbit.

Pathological findings of the exenterated tissue included unidentified and severely necrotic and hemorrhagic tissue.

During the postoperative period, the patient remained under intensive supervision for wound infections and possibly missed larvae. Vaccination for *Clostridium tetani* was performed. As the wound began to heal, the patient’s general condition improved and she was discharged after two weeks.

## DISCUSSION

Diptera or true flies are a large order containing an estimated number of 240,000 insects.^1^ The life cycle of flies includes eggs (which may be present in clumps of up to 300), larvae, pre-pupa, pupa, and adult flies. The larva or maggot is the main feeding stage, during which the insect grows from 2 mm to 15–20 mm in length in approximately 4 days.^2^

The larvae of some Diptera species are obligatory parasites and cannot survive outside animal tissues or organs; others may be facultative and can live both on and outside animal tissues.^3^ Some diptera species that cause myiasis, such as *Oestrus ovis* or botfly, are larviparous and inject their larvae into the eyes, nostrils, and nasopharynx of sheep and goats, and occasionally infest humans. Other diptera species are oviparous and lay eggs on peripheral wounds and wound debris or on the discharge around fistulae. The eggs hatch and the larvae can penetrate the wounds, feeding on living or necrotic tissue.^4^

Myiasis is defined as infestation of live human and animal tissues or organs by Diptera larvae. Depending on the involved site, myiasis may be called ophthalmomyiasis, otomyiasis, or nasopharyngeal, cutaneous, vaginal, anal, urogenital, and intestinal myiasis.

*Dermatobia hominis*, endemic to tropical or subtropical areas, and *Oestrus ovis* (sheep botfly) cause most cases of ophthalmomyiasis.^5^

Since the first published case^6^ of ophthalmomyiasis due to *Oestrus ovis* in 1977, several cases have been reported from Iran. Since a vast area of Iran is located in subtropical regions and most rural residents of these regions are in close contact with domestic animals such as goats and sheep, the actual number of cases may be far more than that reported.

The most common presentation of ocular myiasis is external ophthalmomyiasis, a condition which presents with conjunctivitis-like symptoms such as itching, foreign body sensation, and chemosis. Masoodi and Hosseini^7^ reported eight farmers from Fars province, Iran with external ophthalmomyiasis, in whom all symptoms resolved following removal of the larvae from the bulbar conjunctiva. The number of larvae in these cases ranged from one to five and the larvae were identified as first instars of *Oestrus ovis* (Diptera: Oestridae) which is a larviparous diptera.

Larvae may penetrate the sclera and migrate into the eye, where they can cause ophthalmomyiasis interna.^8^ There have been two such cases reported from Iran. A case of bilateral subretinal ophthalmomyiasis interna was reported in a 70-year-old woman in 1997. Diagnosis was based on subretinal migratory tracks on fundus examination and fluorescein angiography.^9^ The second case was a 12-year-old boy with anterior ophthalmomyiasis interna, in whom the larva initially presented in the anterior chamber but migrated after 3 days through a peripheral iridectomy (created by the larva itself) into the posterior chamber. The larva was removed by pars plana vitrectomy and identified as a blowfly (Diptera: Calliphoridae).^10^

It seems that underlying disorders such as skin cancer (basal or squamous cell carcinoma) or neurological and metabolic disorders (such as diabetes mellitus), as well as poor hygiene may be risk factors for myiasis, probably due to increased susceptibility to non-healing wounds.^4^,^11^–^14^

In most reports the number of larvae has been few.^8^–^11^,^15^ To our knowledge the only case of ophthalmomyiasis with massive infestation reported from Iran was in Khuzestan.^4^ The patient described herein is the second. Both cases were caused by *Chrysomya bezziana* in elderly patients with underlying diseases.

The first case of ophthalmomyiasis due to *Chrysomya bezziana* was reported in 1986 in a 65-year-old man, who underwent exenteration due to severe involvement of orbital tissues.^16^ The diagnosis of *Chrysomya bezziana* is based on the form of the spiracles, cephalopharyngeal skeleton, and the shape and size of developed larvae: the posterior spiracles do not have a distinct button and the number of lobes on the anterior spiracles is six. The robust spine bands constitute another identifying feature of *Chrysomya bezziana*.^15^

Treatment consists of removal of the larvae. Surgical debridement under local anesthesia is curative, although a foreign body reaction may occur if parts of the larvae remain. If the number of larvae is few occlusion-suffocation approaches, including petroleum jelly, liquid paraffin, beeswax, nail polish, or heavy oil placed over the central area, seem to be effective. Such blockage forces the aerobic larvae to surface for air, at which time they can be captured by forceps. Additionally, lidocaine can be injected into the base of the cavity in which the larvae inhabit, forcing the larva to the surface. Alternatively, ethyl chloride sprays, liquid nitrogen, and insecticides have been used alone or in combination.^17^

There are few reported cases of ophthalmomyiasis severe enough to necessitate exenteration.^4^,^13^,^16^ Our patient is the second case of massive ophthalmomyiasis in Iran leading to exenteration. Managing the wound during the postoperative period is extremely important; these patients are usually in poor general health conditions, therefore antiseptic dressings and antibiotics are frequently indicated. Myiasis can serve as a portal of entry for *Clostridium tetani* and vaccination should be considered in affected individuals.^17^

Ivermectin is effective for both prophylaxis and treatment of botfly infestations in livestock. Ivermectin may be beneficial as adjunctive therapy in select cases of ophthalmomyiasis, but concrete evidence is lacking in this regard.^5^ There has been one report of ophthalmomyiasis externa caused by *Dermatobia hominis* treated with ivermectin.^18^

Although there is no absolute method for protection against myiasis, prevention could consist of practicing adequate personal hygiene, proper care of wounds and treatment of debilitating underlying conditions.
